# Factors influencing inequality in government health expenditures within African regional economic communities

**DOI:** 10.1186/s12913-024-10783-w

**Published:** 2024-03-07

**Authors:** Nicholas Ngepah, Ariane Ephemia Ndzignat Mouteyica

**Affiliations:** School of Economics, College of Business and Economics, University of Johannesburg, Johannesburg, South Africa

**Keywords:** Health expenditure, Inequality, Linear mixed-effect approach, RECs, Africa

## Abstract

**Background:**

The unequal distribution of government health spending within African regional economic groupings is a significant barrier to achieving Universal Health Coverage and reaching health-related Sustainable Development targets. It also hampers the progress toward achieving the African Union’s vision of an integrated and prosperous Africa, free of its heavy disease burden. Based on panel data from 36 countries nested into eight Regional Economic Communities (RECs), this study probes the effects of countries' macro-level factors on government health expenditure disparities within eight regional economic communities from 2000 to 2019.

**Method:**

We use the multilevel linear mixed-effect method to show whether countries' trade gains, life expectancy at birth, poverty, urbanization, information and communication technology, and population aging worsen or reduce the differences for two government health expenditure indicators.

**Results:**

The insignificant effect of GDP per capita suggests that in most regional economic groupings, the health sector is still not considered a high-priority sector regarding overall government expenditures. Countries' poverty levels and urbanization increase the domestic general government health expenditure disparities as a percentage of general government expenditure within the regional groupings. However, trade gains and ICT diffusion reduce these disparities. Furthermore, the results reveal that external health expenditure per capita and life expectancy at birth positively impact within-regional inequalities in the domestic general government health expenditure per capita. In contrast, GDP per capita and trade gains tend to reduce them.

**Conclusions:**

This study enriches the research on the determinants of government health expenditure inequality in Africa. Policies that can spur growth in trade and ICT access should be encouraged. Countries should also make more efforts to reduce poverty. Governments should also develop policies promoting economic growth and planned urbanization.

**Supplementary Information:**

The online version contains supplementary material available at 10.1186/s12913-024-10783-w.

## Introduction

Health expenditure in Africa remains insufficient to meet the growing healthcare financial needs caused by communicable and non-communicable diseases [1]. The region faces a significant financial gap in achieving the health targets set in the Sustainable Development Goals (SDGs) of the 2030 Agenda for the UN Sustainable Development, with an estimated annual average shortfall of US$66 billion [2]. Furthermore, declining official development assistance per capita adds strain to the region's healthcare funding. Over the years, numerous initiatives and policies have aimed to address these challenges and enhance the health and well-being of the African populations [3]. These efforts involve regional, continental, and global commitments to strengthen health systems, boost health investments, address social determinants of health, and enhance equity across countries and regions. The 2001 Abuja Declaration, calling for African governments to allocate at least 15 percent of national budgets to health, is one of key aspects of these policy frameworks [3]. The declaration urges signatory countries to prioritize government health investments and calls on donor countries to increase health funding levels to support these endeavors [4].

The common initiatives and policies among African governments create a comprehensive and flexible platform, facilitating countries to converge and enhance coherence for improved health outcomes in Africa and within African Regional Economic Communities (RECs). However, progress on these joint health commitments has been insufficient to address weaknesses in health systems and disparities in government health expenditures, posing significant barriers to achieving the SDGs and Universal Health Coverage (UHC) in RECs [3, 5]. Adebisi et al. [6] showed that the disparities in government health expenditures stem from differences in the degree of prioritization of health by African governments, particularly evident in ECOWAS countries with lower health sector investments compared to the defense sector and insufficient funding in sectors crucial for health impact, such as social protection and disaster response. Additionally, the COVID-19 pandemic, coupled with challenges like high public indebtedness, climate pressure, the war in Ukraine, and competing national priorities, is deteriorating macroeconomic conditions. These challenges further constrain countries' ability to increase public health revenues, exacerbating disparities in government health spending within and between countries [7]. Moreover, mounting inflation pressure imposes limitations on aid budgets, posing challenges for developing countries, especially African nations, to receive necessary external assistance [4].

Previous studies have primarily focused on the driving forces behind the variations in public health spending in OECD and European Union (EU) countries, studies in the African context remain rare. Additionally, most of these existing studies used micro-level data. However, it is essential to incorporate macro-level analysis to guide policymaking. Relying solely on the micro-level analysis may be counterproductive, exacerbating the problem, as policymakers might not consider the broader aggregate perspective [4]. Furthermore, there are gaps in the literature regarding the impacts of country-specific characteristics on disparities in government health expenditures within the African regional economic communities. Boutaleb, [8] showed that many African countries experience significant socioeconomic, environmental, and political challenges that worsen health expenditure inequality in the RECs and hinder deeper and successful regional health integration. These challenges include corruption, poor governance, persistent conflicts, and insecurity. Additionally, African Union, [3] highlighted countries' crucial role in generating sustainable public financial resources and prioritizing health by considering the Abuja Declaration target and the recommendations of the World Health Organization (WHO) and the High-level Taskforce on Innovative International Financing for Health Systems (HLTF). The report also emphasized the role of governments in adopting and incorporating the existing regional strategic plans and policies on health financing.

Therefore, empirically examining the impact of countries' macro-level characteristics on government health expenditure disparities within the African regional groupings is necessary to guide policymakers to develop policies that promote deeper health integration, essential for improving health outcomes and attaining convergence at the regional and continental levels. This study aims to fill this gap by investigating how countries’ macro-level factors influence disparities for two government health expenditure indicators in eight African RECs. These indicators include the domestic general government health expenditure as a percentage of general government expenditure (the so-called Abuja policy instrument) and the domestic general government health expenditure per capita. The paper further adds to the literature on the field in that it uses the multilevel linear (ML) mixed-effect model. This method is suitable for analysing non-independent and hierarchical data at multiple levels. In this case, 36 African countries are nested into the eight regional economic communities recognised by the African Union. It also considers fixed and random effects and accommodates repeated measurements. The standard methods used in the previous studies, such as the Ordinary Least Square (OLS) and fixed effect approaches, have limitations due to their reliance on the assumption of independent observations, making the multilevel model more appropriate for this study [9].

The outline of this paper is as follows: Section 2 presents the existing literature on health expenditure inequality. Section 3 describes the methods adopted and the data used. Section 4 provides the empirical results and their discussion. Section 5 provides the conclusion and policy recommendations.

## Existing literature on health expenditure inequality

Previous studies used various econometric techniques to identify the determinants of health expenditure inequality. They distinguish between macro- and micro-level explanations [10, 11]. For instance, several studies revealed that income is a significant driver of cross-country differences in health expenditure level and growth [12]. However, other studies criticized the possible non-stationarity of health spending and income. Additionally, income elasticities are too small when using regional-level data [13]. Other non-income determinants of cross-country health expenditure inequality include age structure, technological progress, and institutional factors. For instance, the younger population's share is regarded as an important indicator of health expenditure variations. However, little empirical evidence exists on the significant impact of this indicator. In most studies, the aging population has been given particular attention, especially in Europe and OECD countries [14, 15].

Additionally, since the work of Newhouse, [16], technological progress has been identified as crucial for health expenditure growth. Studies used several proxies for changes in medical care technology. In cross-section studies, such proxies include surgical procedures and the number of specific medical equipment [17], life expectancy, and infant mortality [15]. These studies concluded that technological progress and variations in medical practices determine the level and growth of health expenditure in OEDC countries. However, the effect of technological progress on the differences in health expenditures within and between countries has not yet been widely empirically tested for non-OEDC countries due to the lack of reliable data. Moreover, very few studies found evidence of a relationship between intuitional factors and the levels of health expenditures [18]. For instance, Studies on OEDC countries showed that health expenditure per capita was higher in countries with a social health expenditure mechanism [19]. Other studies at the macro-level found that factors aggregate population health [20] and health aid significantly impact cross-country health expenditure disparities [21]. Furthermore, there is an ongoing discussion on whether macroeconomic factors like trade, ICT, and GDP per capita also affect the inequalities in health expenditure. A report from [4] showed that worsening macroeconomic conditions influence countries' ability to meet health financing commitments and policies, exacerbating disparities within and across regions.

Furthermore, several empirical approaches for modelling the determinants and variations in health expenditures are presented in the literature. Some studies relied on cross-sectional techniques, while others adopted panel techniques. For instance, Chou and Wang, [22] examined the relationship between health expenditure inequality, income inequality, and provincial government budget deficit (BD). They used new panel cointegration tests with expenditure data for urban and rural areas in China. Their results indicated that income inequality and real government BD significantly explain within-country health expenditure disparities. Ke et al. [23] employed standard fixed effects and dynamic models to identify the factors determining the growth of health expenditures, government health expenditures, and out-of-pocket payments in a panel of 143 countries from 1995 to 2008. Their finding showed a significant variation in health expenditure as a share of GDP across countries. The factors contributing to this variation include income, demographics, and health system characteristics. The authors also found that government health expenditures and out-of-pocket payments follow different paths. In contrast, the pace of health expenditure growth is different for countries at varying levels of economic development. Nghiem and Connelly, [24] recently focused on the trend and determinants of health expenditure in OECD countries from 1975 to 2004. They hypothesized that health expenditure is a function of the aging population, technological progress, and health insurance. The results suggest that the main driver of health expenditure is technological progress.

Recently, Behera and Dash [25] examined the impact of macro-fiscal policies on health financing in 85 low- and middle-income countries from 2000 to 2013. They used the panel System Generalized Method of Moment model that captures the endogeneity problem in the regression estimation. They divided the LMICs into four sub-samples: pre-global financial crisis (2000–2008), post-global financial crisis (2009–2013), low-income, and middle-income. They found that tax revenue, aging, and per capita income positively affect public health expenditure in the full sample, while the impact of debt service payments is negative. Moreover, they found significant variations in the coefficient values for the sub-samples. More recently, Micah et al. [26] employed the Shapley decomposition to investigate the determinants and variations of government health spending in 46 Sub-Saharan countries. They found a significant positive relationship between government health spending and good governance. They also found substantial variations in government health spending across the countries.

Following the literature, a few studies used macro-level data to investigate the variations in health expenditure within regional economic arrangements. Most of these studies focused on OECD and EU countries. In Africa, these studies are rare. In addition, no study has attempted to examine whether country-level characteristics influence inequality in government health expenditure within African regional economic grouping. Therefore, this study focuses on the role of African countries' GDP per capita, poverty, urbanization, external assistance for health, population structure, trade, ICT, and disease burdens on two domestic general government health expenditure indicators within the eight RECs recognized by the African Union. These include the Arab Maghreb Union (UMA), the Common Market for Eastern and Southern Africa (COMESA), the Community of Sahel–Saharan States (CEN–SAD), the East African Community (EAC), the Economic Community of Central African States (ECCAS), the Economic Community of West African States (ECOWAS), the Intergovernmental Authority on Development (IGAD), and the Southern African Development Community (SADC).

The study also uses the multilevel linear mixed-effect model, composed of fixed and random effect components. The model is appropriate for hierarchical data. The method also allows the analysis of repeated measurements [9]. In contrast to standard techniques such as fixed effect and Ordinary Least Square OLS, the multilevel linear mixed-effect model allows the analysis of non-independent data organized at multiple levels. This study has two levels: the countries represent the lower level, and the RECs represent the higher one. Additionally, when modelling clustered data and multiple levels of clustering, ML mixed-effect approach is more appropriate because it can account for the correlation among observations at different levels of data compared to approaches such as GMM that only account for clustered data at one particular level [27]. Moreover, ML mixed-effects models have been employed in various fields to address endogeneity concerns in the context of nested or clustered data. The approach includes a combination of fixed and random effects in which the random effects account for the correlation among the measurements per subject at different time points [28].

## Method

### Model specification

Deriving from the literature on the macro-level determinants of health expenditure inequality, we examine how country-specific characteristics affect health expenditure disparities among eight African regional economic communities. To this end, we use the multilevel linear mixed-effect model composed of fixed and random effects. The model is as follows:

1 $$y=X\beta +Z\mu +\epsilon$$

The variable $y$ represents an $n \times 1$ column vector of response, also known as the outcome variable and mean $E\left(y\right)=X\beta$; $X$ represents an $n \times p$  design matrix of the $p$ predictor variables, which is also known as the covariate matrix of the fixed effects component;$\beta$ represents a $\rho \times 1$ column vector of fixed effects regression coefficients ($the \beta s)$; ${Z}_{i}$(with $Z=[{Z}_{1}, \dots , {Z}_{b}]$) is an $n \times {q}_{i}$ design/covariate matrix for the q random effects component, while $i$ represents the groups. $\mu$ represents the vector of random effects ($q \times 1)$, with a variance-covariance matrix $Q$ and orthogonal to $\epsilon$ such that $$Var\;\begin{bmatrix}\mu\\\epsilon\end{bmatrix}=\begin{bmatrix}Q&0\\0&\vartheta_\epsilon^2H\end{bmatrix}$$; $\epsilon$ represents a vector of the residuals ($n \times 1$), with $E\left(\epsilon \right)=0$ and variance matrix is ${\vartheta }_{\epsilon }^{2}H$. We use the general forms of the design matrices $X$ and $Z$ because they allow a flexible modeling approach to within-panel correlation. Countries in the same panel are correlated due to a common random intercept or a common random slope.

The Multilevel linear mixed-effect model is appropriate for estimating the variance components compared to standard methods such as ANOVA, Fixed effect, and Pooled regression, which are subject to several flaws. For instance, the ANOVA method is challenging to apply in unbalanced data or more complex variance structures [29]. The minimum norm quadratic unbiased estimation (MINQUE) was suggested by Rao, [30] to estimate the variance parameters when dealing with unbalanced data. At the same time, LaMotte, [31] recommended using the minimum variance quadratic unbiased estimation (MIVQUE) for the same purpose.

However, the maximum and residual maximum likelihood approaches have been commonly used to estimate the variance parameters for balanced and unbalanced data. The methods are appropriate for a more comprehensive class of variance models than the simple variance components. In the current study, we adopt the maximum likelihood estimates deriving from the likelihood theory. In our panel data, the multilevel linear mixed-effect model is organized as a series of $W$-independent panels instead of accounting for all $n$ observations. The model is as follows:

2 $${y}_{i}={X}_{i}\beta + {Z}_{i}{\mu }_{i}+{\epsilon }_{i}$$

Where $i$ is a panel of ${n}_{i}$ observations, such that $i=1, \dots , W$; ${y}_{i}$ represents the response, composed of the rows of $y$ corresponding to the $ith$ panel. ${X}_{i}$ and ${\epsilon }_{i}$ are analogously defined; ${\mu }_{i}$ represents the $W$ realization of a $q \times 1$ vector. ${\mu }_{i}$ is normally distributed, with a mean equal to zero and a $q \times q$ variance matrix Σ; ${Z}_{i}$ represents a ${n}_{i} \times q$ design matrix for the $ith$ panel random effects. When considering Eq. (1), we can write the following:

3 $$Z=\begin{bmatrix}Z_1&0&\dots&0\\0&Z_2&\dots&0\\\vdots&\vdots&\ddots&\vdots\\0&0&0&Z_W\end{bmatrix};\mu=\begin{bmatrix}\mu_1\\\vdots\\\mu_W\end{bmatrix};Q=I_W\otimes\Sigma;H=I_W\otimes\Lambda$$

The model presented in Eq. (2) was proposed by Laird and Ware, [32] to allow an easy specification of the random effect component. This formulation can also lead to more than one level of random variation. Equation (2), a one-level model, can be expanded to more levels. This study has two levels, with countries being nested within regional economic communities. The regional economic groupings are level one, and countries are level two. We assume that residuals have constant variance and are independent.

### Data

The dataset used in this study is extracted from the World Bank Development Indicators (WDI), the World Governance Indicators (WGI), the World Health Organization (WHO), and PovcalNet. The empirical analysis covers a panel of 37 African countries and eight African regional economic communities from 2000 to 2019. The countries were selected based on data availability; hence, we did not have issues with missing data. The Gini coefficients of the domestic general government health expenditure as a percentage of general government expenditure (extracted from the WDI) and domestic general government health expenditure per capita, PPP (extracted from the WHO) are the dependent variables. These two variables are computed at the regional level, while the independent variables are obtained at the country level. We took the logs of all the variables to reduce data variability. We also used the principal component analysis method to construct governance and ICT indices. The list of countries and variables for the study is presented in Appendix 1. The analyses were done using Stata 16 software.

## Empirical results

### Correlation matrix and principal component analysis tests

Panels A1 and B1 of Table 1 reveal the correlation matrix results. In Panel A1 of Table 1, we show the correlation between six governance indicators: government effectiveness, political stability and absence of violence/terrorism, control of corruption, the rule of law, voice and accountability, and regulatory quality. However, in Panel B1 of Table 1, we used two ICT indicators: the individual using the internet and mobile cellular subscriptions. The results reveal high collinearity in both cases. Given the correlation results, we employed the principal component analysis (PCA) to construct the governance and ICT indices. The indices allow us to investigate the impact of governance and ICT on health outcome disparities across RECs. The PCA results reported in Panels A2 and B2 show that component 1 is preferable to the other components because the eigenvectors linked to the variables whose loading exceed 0.4 in absolute term is less than its eigenvalue for both indices [33].

**Table 1:** Correlation matrix and principal component analysis results

**Panel A1: Governance indicators' correlation matrix results**							
	**Effectiveness**	**Stability**	**Corruption**	**Regulation**	**Law**	**Accountability**	
Effectiveness	1.000						
Stability	0.647	1.000					
	0.000						
Corruption	0.824	0.712	1.000				
	0.000	0.000					
Regulation	0.914	0.660	0.783	1.000			
	0.000	0.000	0.000				
Law	0.899	0.772	0.865	0.878	1.000		
	0.000	0.000	0.000	0.000			
Accountability	0.650	0.642	0.635	0.681	0.754	1.000	
	0.000	0.000	0.000	0.000	0.000		
**Panel A2: Principal component results**							
**Components**	**Eigenvalue**	**Difference**	**Proportion**	**Cumulative**			
Component 1	4.787	4.321	0.798	0.798			
Component 2	0.466	0.087	0.078	0.876			
Component 3	0.379	0.168	0.063	0.939			
Component 4	0.211	0.122	0.035	0.974			
Component 5	0.089	0.021	0.015	0.989			
Component 6	0.068		0.011	1.000			
**Panel A3: Principal component eigenvector results**							
**Variables**	**Component 1**	**Component 2**	**Component 3**	**Component 4**	**Component 5**	**Component 6**	**Unexplained**
Effectiveness	0.424	-0.428	0.101	-0.192	-0.380	0.667	0.000
Stability	0.375	0.539	-0.661	-0.327	0.058	0.147	0.000
Corruption	0.412	-0.191	-0.275	0.793	0.294	0.053	0.000
Regulation	0.422	-0.335	0.182	-0.451	0.628	-0.282	0.000
Law	0.442	-0.060	-0.008	0.022	-0.607	-0.658	0.000
Accountability	0.369	0.612	0.667	0.159	0.059	0.135	0.000
**Panel B1: ITC indicators correlation matrix results**							
	**Internet**	**Mobile**					
Internet	1.000						
Mobile	0.812	1.000					
	0.000						
**Panel B2: Principal Component results**							
**Components**	**Eigenvalue**	**Difference**	**Proportion**	**Cumulative**			
Component 1	1.812	1.624	0.906	0.906			
Component 2	0.188		0.094	1.000			
**Panel B3: Principal component eigenvector results**							
**Variables**	**Component 1**	**Component 2**	**Unexplained**				
Internet	0.707	0.707	0.000				
Mobile	0.707	-0.707	0.000				

**Source:** Author’s computation

### Descriptive statistics

Table 2 reports the descriptive statistics of the variables used. The average Gini coefficient of government health expenditure (the domestic general government health expenditure (as a percentage of GGE) was approximately 0.23, ranging from 0.01 to 0.36. On average, the Gini coefficient of domestic health expenditure (which represents the domestic general government health expenditure per capita) was 0.63, with a minimum of 0.05 and a maximum of 0.85. These results suggest moderate to high cross-regional inequalities in health expenditure in African RECs from 2000 to 2019.

**Table 2:** Descriptive statistics of variables of the full sample

**Variables**	**Mean**	**Std. Dev.**	**Min**	**Max**
**Dependent variables**				
Gini government health exp.	0.229	0.050	0.006	0.357
Gini domestic health exp. cap	0.630	0.137	0.051	0.851
**Independent variables**				
GDP per capita	4100.463	3872.706	715.454	22869.760
POP above 65	3.279	1.410	1.871	11.999
POP below 15	41.920	6.429	17.260	50.264
Urban population	38.555	16.129	8.246	89.741
Poverty	0.396	0.237	0.001	0.928
HIV	1.922	3.284	0.010	21.680
External health exp.	26.010	30.373	0.124	223.979
Life expectancy	58.778	7.612	39.441	76.880
Non-communicable diseases	24.888	5.490	13.900	47.900
Governance	4.481	2.188	0.000	10.558
Information and communication technology	1.310	1.346	0.000	6.155
Trade	61.462	27.046	1.219	175.798
Debt services	2.912	4.140	0.053	46.340
Foreign Direct Investment	3.166	4.379	-11.197	46.275

Source: Authors’ computation from WDI, WGI, and PovcalNet, WHO datasets

On average, GDP per capita was about US$4100.46, with a minimum of US$715.45 and a maximum of US$22869.76. There is evidence of variabilities across countries, as shown by the standard deviation of US$3872.71. In Africa, approximately 41.92 percent of the total population aged 15 years and younger, and less than four percent were above 65, indicating a low life expectancy at birth, estimated at 58.78 years in the region. These findings are consistent with Kaba, [34], who showed that Africa has many young people. Approximately 38.56 percent of African people lived in urban areas, with a maximum of 89.74 percent. An average of 0.40 percent of people live in poverty (headcount ratio). The average external health expenditure per capita was US$26.01, with a maximum of US$223.98. Regarding the disease burden, about 24.99 percent of people aged 30 to 70 died from CVD, cancer, diabetes, and CRD. The average HIV incidence in the continent was about 1.92 per 1,000 uninfected people. Trade represented 61.46 percent of GDP between 2000 and 2019. On average, only 1.31 percent of the African population has access to ITC, and the governance indicator was 4.49. Additionally, the total debt services accounted for 2.91 percent of the gross national income during the study period, while foreign direct investment represented 3.17 percent of the GDP.

We also report the summary statistics of the disparities in government health expenditure indicators within and between RECs in Table 3. The results reveal that inequality in the share of government health expenditure to government expenditure was higher in the ECCAS region (0.29), followed by SEN-SAD (0.24), SADC (0.24), and ECOWAS (0.23). The IGAD region appeared to be relatively equal regarding the variable of interest. In the ECCAS region, countries like Congo, DRC, and the Central African Republic had shares of government health expenditure to government expenditures below 5 percent. In contrast, countries like Burundi and Chad spent closer to 10 percent of their budget on health, as Micah et al. [26] reported. Additionally, [8] showed that the level of prioritization of health by members of the ECOWAS and SADC regions is low. Only three SADC countries (Seychelles, Botswana, and Eswatini) allocated over 15 percent of their national budgets to health. However, no government in ECOWAS reached the target between 2001 and 2019.

**Table 3:** Summary statistics of the levels of government health expenditure inequality within the RECs

	General Government health expenditure as a percentage of general government expenditure				Domestic general government health expenditure per capita			
REC	Mean	Std. Dev.	Minimum	Maximum	Mean	Std. Dev.	Minimum	Maximum
UMA	0.205	0.045	0.136	0.280	0.382	0.101	0.051	0.529
COMESA	0.218	0.040	0.156	0.297	0.709	0.073	0.544	0.851
EAC	0.172	0.022	0.139	0.206	0.639	0.082	0.375	0.732
ECCAS	0.290	0.039	0.215	0.357	0.602	0.122	0.422	0.824
ECOWAS	0.232	0.028	0.179	0.280	0.616	0.130	0.303	0.831
IGAD	0.105	0.067	0.006	0.283	0.316	0.145	0.127	0.647
SADC	0.236	0.028	0.186	0.288	0.625	0.086	0.506	0.851
SEN-SAD	0.242	0.027	0.184	0.299	0.702	0.066	0.617	0.842
Total	0.229	0.050	0.006	0.357	0.630	0.137	0.051	0.851

Source: Authors’ computation from the World Development Indicators

In contrast, there is high inequality in per capita government health expenditure. This inequality is well-pronounced in COMESA (0.71), followed by SEN-SAD (0.70). The EAC, SADC, and ECOWAS regions also had high inequality for the variable of interest, with averages above 0.60. However, moderate inequality was recorded in the UMA and IGAD regions. Micah et al. [26] also reported significant differences in countries' per capita government health spending.

### Multilevel mixed-effects linear regression results

We first performed the intra-class Correlation test, which is reported in Table 4. This test allows us to verify whether the multilevel linear mixed-effect model is appropriate for this study. According to Hecker et al. [35], the approach is suitable when the ICC value is above zero. The ICC values are 0.55 and 0.53 for the domestic general government health expenditure (as a percentage of GGE) and the domestic general government health expenditure per capita. These results suggest that the selected model is suitable for the current study.

**Table 4:** Residual intra-class correlation

Variables	government health exp. model	Domestic health exp. cap model
ICC	0.549	0.525
Standard deviation	0.046	0.046

Source: Authors' computation

Table 5 presents the empirical results. The first Columns list the explanatory variables used in the study. The multilevel linear mixed-effect regression results for the two variables of interest are reported in the second and fifth columns. The third and sixth columns report results from the fixed effect model results. The fourth and seventh columns illustrate the pooled regression model results. The fixed effect and pooled regression models are used for robustness checks. The multilevel linear mixed-effect regression results are robust and satisfactory. The regressions have Chi2 values of zero (Prob>Chi2 = 0.0000), presenting a good fit for the regression model.

**Table 5:** Multilevel linear mixed effect results

	General Government health expenditure as a percentage of the general government expenditure model			Domestic general government health expenditure per capita model		
	**(1)**	**(2)**	**(3)**	**(1)**	**(2)**	**(3)**
	**MLM effect model**	**Fixed effect model**	**Pooled regression model**	**MLM effect model**	**Fixed effect model**	**Pooled regression model**
**Variable**	**robust**	**robust**	**robust**	**robust**	**robust**	**robust**
Log GDP per capita	-0.038	-0.005	-0.058^a^	-0.077^c^	-0.005	-0.058^a^
	(0.043)	(0.074)	(0.019)	(0.043)	(0.074)	(0.019)
Log POP above 65	0.073	-0.015	0.367^a^	0.082	-0.015	0.367^a^
	(0.083)	(0.100)	(0.062)	(0.085)	(0.100)	(0.062)
Log POP below 15	0.047	0.036	0.510^a^	-0.076	0.036	0.510^a^
	(0.163)	(0.192)	(0.134)	(0.166)	(0.192)	(0.134)
Log urban population	0.198^a^	0.062	0.137^a^	0.050	0.062	0.137^a^
	(0.061)	(0.126)	(0.026)	(0.061)	(0.126)	(0.026)
Log poverty	0.023^a^	0.021^a^	0.018^b^	0.005	0.021^a^	0.018^b^
	(0.007)	(0.008)	(0.008)	(0.008)	(0.008)	(0.008)
Log HIV	-0.004	-0.004	-0.008^a^	-0.003	-0.004	-0.008^a^
	(0.003)	(0.003)	(0.003)	(0.003)	(0.003)	(0.003)
Log external health exp. cap	-0.000	-0.000	-0.009	0.017^c^	-0.000	-0.009
	(0.009)	(0.010)	(0.009)	(0.009)	(0.010)	(0.009)
Log life expectancy	0.244	0.413^b^	-0.189	0.346^b^	0.413^b^	-0.189
	(0.165)	(0.207)	(0.124)	(0.168)	(0.207)	(0.124)
Log Non-communicable disease	0.032	-0.066	0.110^b^	0.113	-0.066	0.110^b^
	(0.090)	(0.115)	(0.050)	(0.091)	(0.115)	(0.050)
Log governance	0.028	0.005	0.016	-0.032	0.005	0.016
	(0.029)	(0.035)	(0.019)	(0.030)	(0.035)	(0.019)
Log Information and communication technologies	-0.038^a^	-0.045^a^	-0.006	0.003	-0.045^a^	-0.006
	(0.008)	(0.009)	(0.008)	(0.008)	(0.009)	(0.008)
Log trade	-0.083^a^	-0.116^a^	0.039^b^	-0.045^b^	-0.116^a^	0.039^b^
	(0.019)	(0.021)	(0.017)	(0.019)	(0.021)	(0.017)
Log Foreign Direct Investment	0.009	0.010^c^	-0.005	0.004	0.010^c^	-0.005
	(0.006)	(0.006)	(0.006)	(0.006)	(0.006)	(0.006)
Log debt services	0.009	0.006	0.028^a^	0.004	0.006	0.028^a^
	(0.008)	(0.008)	(0.009)	(0.008)	(0.008)	(0.009)
Constant	-2.973^a^	-2.824^b^	-3.579^a^	-1.456	-2.824^b^	-3.579^a^
	(1.136)	(1.296)	(0.965)	(1.160)	(1.296)	(0.965)
Observations	1,394	1,394	1,394	1,394	1,394	1,394
R-squared			0.146			0.146
Prob > F		0.0000			0.0000	
Prob > chibar2	0.0000			0.0000		
Prob >Chi2	0.0000			0.0000		

^a^, ^b^, and ^c^ symbolizes significance at 1 percent, 5 percent, and 10 percent, respectively. The standard errors are in parentheses. Source: Authors' computation. Data retrieved from the World Development Indicators and the World Governance Indicators

#### The domestic general government health expenditure (as a percentage of GGE) results

The multilevel linear mixed-effect regression results are reported in Column 2 of Table 5. The empirical results reveal that countries’ levels of GDP per capita, HIV incidence, external health expenditure per capita, governance, life expectancy at birth, and their share of the population aged 15 and below 65, death associated with non-communicable diseases, foreign direct investment, and total debt services are statistically insignificant in explaining disparities in the Abuja Declaration instrument. Out of the 14 variables presented in the model, only four were statistically significant to affect inequalities in the domestic general government health expenditure (as a percentage of GDP) (also known as the Abuja Declaration instrument) in the African Regional Economic Communities. These include countries' poverty levels, urbanization, trade gains, and ICT diffusion. For instance, the positive signs of poverty and urbanization suggest that a one-unit increase in countries' poverty levels and urban population positively influence within RECs inequalities in the domestic general government health expenditure (as a percentage of GGE) by 0.02 and 0.20 log points, respectively. However, the empirical results indicate that an increase in countries' gains from trade and ICT diffusion decreases these inequalities by 0.08 and 0.04 log points.

#### The domestic general government health expenditure per capita results

As Column 5 of Table 5 reports, only four variables presented in the model are statistically significant at 1 and 5 percent in influencing per capita domestic general government health expenditure inequality within the African regional economic grouping. The results also show that a unit increase in countries' GDP per cap reduces within-regional disparities in per capita domestic general government health expenditure by about 0.08 log points. In contrast, an increase in countries' life expectancy at birth and external health expenditure per capita increases these inequalities by 0.35 and 0.02 log points. The empirical results also reveal that increased countries' gains from trade decrease the within-regional variations in the variable of interest by 0.05 log points. Countries’ macro-level factors such as governance index, HIV incidence, the share of population below 15 years old and above 65 years old, poverty level, urbanization, and the burden resulting from non-communicable diseases,the share of foreign direct investment in GDP, the share of total debt services in gross national income, and ICT have an insignificant impact on the variable of interest in the RECs.

#### Robustness of estimates

We performed the fixed effect and pooled regression to test the robustness of our results. The findings in Columns 6 and 7 of Table 5 show that the two models significantly affect the signs and magnitudes of the estimated coefficients. The results from the fixed-effect models are relatively similar to the multilevel linear mixed-effect in terms of the significance and signs in most cases. The estimated coefficients are also fairly close. However, there are substantial differences when considering the pooled regression results.

## Discussion

The evidence of inequalities in health expenditures in the African countries and regional economic groupings is well-established in the literature [36]. However, little is known about the factors that affect such inequalities. This paper investigated the impacts of country-level characteristics on disparities in general government health expenditure (as a percentage of GGE) and general government health expenditure per capita in eight African Regional Economic Groupings. Our empirical findings confirm the impacts of country-level characteristics on within-regional public health expenditure disparities. The results suggest that countries' level of ICT diffusion significantly reduces differences in within-regional government health expenditure (as a percentage of GGE). Increased countries' ICT diffusion negatively affects within-regional health expenditure inequality through enhanced monitoring and diseased surveillance systems, improved data quality and availability, and improved health data management among countries [37]. Rana et al. [38] also found that increasing ICT diffusion significantly affects health expenditure, reducing disparities across countries.

Moreover, the findings suggest that increased countries’ trade as a percentage of GDP contribute to reducing within-regional differences in the Abuja Declaration instrument. In this line, Heller [39] found that increased trade gains lead to fiscal space for the health sectors, positively impacting countries' ability to strengthen health systems and meet regional, continental, and international health financing commitments. However, increasing countries' poverty levels and urbanization significantly raise within-regional inequalities in the Abuja Declaration instrument. The positive sign of urbanization is not unexpected considering the unplanned rapid urbanization in several African countries characterized by overcrowding and poor environmental sanitation that stimulate the spread of infectious and non-communicable diseases [40]. Approximately 56 percent of Africa's population lived in slums in 2014, varying across countries. Only 10 percent of Tunisia's urban population lived in slums in 2014. Still, over 90 percent of the urban population in Sudan and the Central African Republic lived in slums during the same year [40]. Moreover, people in African countries are living longer than ever before. The size of the population aged 65 and above increased from 15 million in 1980 to 41 million in 2014. This figure is projected to increase to 150 million in 2050 [2]. The increase in older people significantly affects healthcare financing and provisions among countries, leading to within-regional disparities in public health expenditures [2].

Moreover, WHO, [7] reported that health spending remained highly unequal across countries. In Africa, the share of health in government spending significantly increased among upper-middle-income countries between 2000 and 2011. However, it stagnated in lower-middle countries, whereas there was a decline in the proportion of public health spending in low-income countries. The report also shows that government sources financed most expenditures on health in upper-middle countries. Health prioritization in government spending is low in low and lower-middle-income countries, with high levels of poverty and increased dependence on external aid and out-of-pocket expenditures [7].

Furthermore, our findings suggest that GDP per capita, life expectancy at birth, trade gains, and external health expenditure per capita were statistically significant in influencing disparities in the general government health expenditure per capita. Increased countries' external health expenditure per capita and life expectancy at birth tend to raise within-regional inequalities in the variable of interest. In this line, WHO, [41] showed that patterns of health spending by source vary across income groups. For instance, upper-middle-income countries mostly rely on government sources to finance their health sectors. In contrast, low-income countries heavily rely on external aid and out-of-pocket spending. On the other hand, Dreger and Reimers [15] and You and Okunade [42] have found that life expectancy is a driver of the variations in health expenditure across countries and regions. As a measure of technological changes, life expectancy enhances efficiency. It reveals the differences in the population's overall health status resulting from adopting and providing advanced technology in the health sector. However, in Africa, advanced technology is still in its infancy in most countries, with substantial variations across countries. Low average life expectancy in the region suggests that poor technological capabilities in most African countries remain a significant constraint to addressing within-regional disparities in government health expenditure per capita [43]. The results suggest that countries' trade as a percentage of GDP negatively affect general government health expenditure inequalities per capita. Our findings align with previous studies, such as Kiymaz et al. [44], who found that trade may influence cross-regional disparities in the variable of interest through tariff changes and domestic taxation capabilities.

## Conclusion and policy implications

It is well established in the literature that countries are the principal actors in the regional integration process. The primary responsibilities regarding health policies, including initiating, supporting, and mobilizing resources to improve their populations' health outcomes, still lie with the Member States of the African RECs. Therefore, assessing how countries’ macro-level characteristics can affect disparities in health expenditures in the RECs is necessary to provide informed policies to encourage deeper regional health integration. This paper examined how country-level characteristics affect the differences in public health expenditures within Africa's Regional Economic Communities from 2000 to 2019. Our empirical results reveal that countries' macro-level factors are drivers of government health expenditure disparities in the RECs. Countries' gains from trade, urbanization, poverty level, and ICT diffusion significantly impact the within-regional differences in the Abuja policy instrument. Countries’ trade gains and widespread distribution of ICT are essential for reducing inequalities in the variable of interest in the regional economic groupings. However, countries' poverty levels and urban population tend to increase such disparities.

Additionally, countries’ GDP per capita, life expectancy at birth, trade as a share of GDP, and external health spending per capita are the significant variables affecting within-regional variations in general government health expenditure per capita. Countries' gains from trade and GDP per capita are very beneficial to reducing within-regional differences in the variable of interest. In contrast, countries' life expectancy at birth and external health expenditure per capita tend to worsen such disparities.

The empirical results obtained in this study provide several important policy implications necessary to ensure deeper and successful regional health integration in Africa. Firstly, policies that have the potential to spur growth in trade and ICT diffusion would cause a reduction in the existing disparities in government health expenditures in the RECs. Policymakers should develop policies promoting deeper trade integration within the regional groupings. Moreover, countries should make more efforts toward achieving SDG 1, which aims at reducing poverty. These efforts are crucial, especially given the unprecedented reversal of countries' past efforts to reduce poverty caused by the COVID-19 pandemic. African policymakers should also develop urbanization plans to enhance productivity and living standards in African cities, which is crucial for poverty reduction. These plans should promote planned and regulated urbanization in African cities. The member states of the African RECs should decrease their reliance on donor funding to finance key health interventions. The bulk of funding for health should be mobilized from domestic sources. Thus, it is important to identify new domestic sources to finance health.

Given the impact of GDP per capita in reducing inequalities in the domestic general government health expenditure per capita and amid multiple challenges, including the fallout of COVID-19, the pressure of inflation, and the consequences of the war in Ukraine, countries should encourage policies that stimulate inclusive economic growth. Economic growth should be accompanied by increasing government health expenditure per capita. This can be done through the reallocation of resources from low-to-high-priority sectors. For instance, some resources could be reallocated from the defense sector to the health sector.

However, this study has a few limitations that can be considered for future research. The study did not consider the impacts of significant factors, including countries' inflation rate, climate change proxied by CO2 emissions, countries’fiscal determinants such as tax revenue on within-regional government health spending. Thus, this study can still be improved.

### Supplementary Information

**Supplementary Material 1.** 

## Data Availability

The datasets used in the study are publicly available and can be extracted from the World Bank and World Health Organization database.
