# Supplementary material for: Factors influencing inequality in government health expenditures within African regional economic communities
Source: BMC Health Serv Res. 2024 Mar 7;24:311. doi: 10.1186/s12913-024-10783-w (PMC10921763; doi:10.1186/s12913-024-10783-w)
Supplement: Supplementary file 1 — Supplementary Material 1. [file 12913_2024_10783_MOESM1_ESM.docx]

APPENDIX: LIST OF VARIABLES AND COUNTRIES

| **Panel A: List of variables and description** | | |
| --- | --- | --- |
| Variables | Description | Sources |
| **Dependent variables** | | |
| Government Health exp. | Domestic general government health expenditure (% of general government expenditure) | WDI database |
| Domestic health exp. cap | Domestic general government health expenditure per capita, PPP (constant international US$ | WHO database |
| **Explanatory variables** | | |
| Urban population | Urban population (% of total population) | WDI database |
| POP below 15 | Population ages 0-14 (% of total population) | WDI database |
| POP above 65 | Population ages 65 and above (% of total population) | WDI database |
| Poverty | Headcount ratio | PovcalNet |
| Mobile | Mobile cellular subscriptions (per 100 people) | WDI database |
| Internet | Individual using the internet (% of population) | WDI database |
| HIV | Incidence of HIV, all (per 1,000 uninfected people) | WDI database |
| External health exp. | External health expenditure per capita, PPP (current international $) | WDI database |
| Non-communicable diseases | Mortality from CVD, cancer, diabetes, CRD between exact ages 30 and 70 (%) | WDI database |
| GDP per capita | GDP per capita, PPP (constant 2017 international $) | WDI database |
| Trade | Trade (% of GDP) | WDI database |
| Life expectancy | Life expectancy at birth, all ( | WDI database |
| Effectiveness | Government Effectiveness | WGI database |
| Stability | Political Stability and Absence of Violence/Terrorism | WGI database |
| Corruption | Control of Corruption | WGI database |
| Regulation | Regulatory Quality | WGI database |
| Law | Rule of Law | WGI database |
| Accountability | Voice and Accountability | WGI database |

**Panel B: List countries**

| Algeria | Guinea-Bissau | Tunisia |
| --- | --- | --- |
| Angola | Kenya | Uganda |
| Benin | Madagascar | Zambia |
| Botswana | Mali |  |
| Burkina Faso | Mauritania |  |
| Burundi | Mauritius |  |
| Cameroon | Morocco |  |
| Cabo Verde | Namibia |  |
| Central Afr. Rep. | Niger |  |
| Chad | Nigeria |  |
| Comoros | Rwanda |  |
| Congo, Dem. Rep. | Senegal |  |
| Congo, Rep. | Sierra Leone |  |
| Cote d'Ivoire | South Africa |  |
| Gabon | Sudan |  |
| The Gambia | Swaziland |  |
| Guinea | Togo |  |

**Note**: WDI stands for World Bank's World Development Indicators, while WGI represents for the World Governance Indicators.
